# Eco-friendly sensing of hexavalent chromium ions via copper-doped carbon quantum dots: a fluorescent probe for water safety

**DOI:** 10.1007/s00604-024-06939-4

**Published:** 2025-01-15

**Authors:** Shubam Sudan, Jyotsna Kaushal, Thakur Gurjeet Singh, Mohamed H. Mahmoud, Athanasios Alexiou, Marios Papadakis, Mohammed E. Abo-El Fetoh, Gaber El-Saber Batiha

**Affiliations:** 1 Chitkara University Institute of Engineering and Technology, Chitkara University, Chitkara University, Rajpura, 140401 Punjab India; 2 Centre for Water Sciences, Chitkara College of Pharmacy, Chitkara University, Rajpura, 140401 Punjab India; 3 Chitkara College of Pharmacy, Chitkara University, Rajpura, 140401 Punjab India; 4 School of Public Health, Faculty of Health, University of Technology Sydney, PO Box 123, Broadway, NSW 2007 Australia; 5 Department of Biochemistry, College of Science, King Saud University, Riyadh, 145111 Kingdom of Saudi Arabia; 6 Department of Research & Development, 11741 Funogen, Athens, Attiki Greece; 7 University Centre for Research & Development, Chandigarh University, Chandigarh-Ludhiana Highway, Mohali, Punjab India; 8 University Hospital Witten-Herdecke, University of Witten-Herdecke, Heusnerstrasse 40, 42283 Wuppertal, Germany; 9 Department of Pharmacology and Toxicology, Faculty of Pharmacy, Egyptian Russian University, Badr City, 11829 Cairo Egypt; 10 Department of Pharmacology and Therapeutics, Faculty of Veterinary Medicine, Damanhour University, Damanhour, 22511 AlBeheira Egypt

**Keywords:** Copper-doped carbon quantum dots, Fluorescence sensing, Hexavalent chromium detection, Environmental pollutant monitoring, Hydrothermal synthesis, Selective fluorescent probe

## Abstract

The hydrothermal synthesis is presented of copper-doped carbon dots (Cu-CDs) from citric acid, urea, and copper chloride, resulting in blue-fluorescent particles with stable emission at 438 nm when excited at 340 nm. Through comprehensive spectroscopic and microscopic characterization (FTIR, XPS, UV, and HRTEM), the Cu-CDs demonstrated remarkable stability across varying pH levels, ionic strengths, temperatures, and UV exposure. Notably, Cu-CDs exhibit ultra-sensitive and selective detection of hexavalent chromium [Cr(VI)] ions in aqueous environments driven by fluorescence quenching. The system showed a robust linear response to Cr(VI) in the 0–80 µM range, with an impressive limit of detection (LOD) of 0.186 µM, significantly lower than the WHO’s permissible limit of 0.96 µM. These findings position Cu-CDs as an effective tool for environmental monitoring and water safety applications.

**Graphical Abstract:**

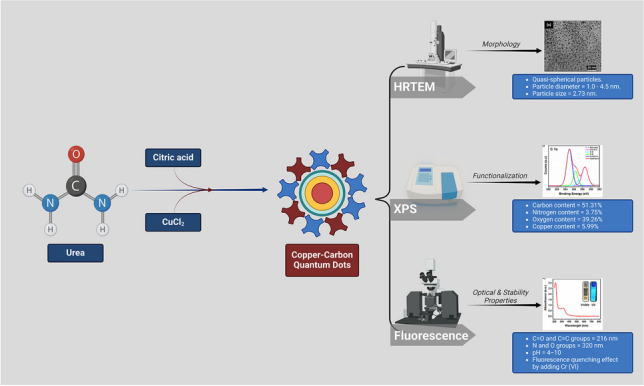

## Introduction

Globally, the rapid rise in the concentration of hazardous metal ions discharged into the environment and the effects on human health have been recognized as severe environmental issues [1–4]. Numerous types of diseases like lung ailments, kidney failure, cancer, schizophrenia, and skin disorders have all been linked to the intake of harmful heavy metal ions [5–7]. Considering this concern, numerous methods have been developed to limit the concentrations of hazardous metal ions in diverse systems [8]. To date, techniques for detecting metal ions at low concentrations (ppm and ppt) have been developed using highly sophisticated instrumentation like mass spectroscopy (MS), X-ray fluorescence spectroscopy (XPS), atomic absorption spectroscopy (AAS), and inductively coupled plasma-mass spectrometry (ICPMS). However, these techniques have several disadvantages, such as low performance, restricted field deployment, high cost, time-intensive procedures, and the requirement for highly qualified operators [9, 10].

Compared to these techniques, the fluorescent probe possesses a wide linear dynamic range, simple sample pretreatment, high sensitivity, fast response, and minimal spectral interference. It has evolved into an established technique for sensing metal ions [11–13]. Fluorescent materials have gained significant attention due to their enormous potential in various sectors, such as chemo/biosensing, bioimaging, optoelectronic devices, and photocatalysis [14–17]. Carbon dots (CDs) have recently emerged as a conspicuous area of study in the context of metal ion sensing at the national and international levels [18]. In the past few years, CDs have frequently been used to sense toxic metal ions like Ni^2+^, Co^2+^, Fe^3+^, Zn^2+^, Cd^2+^, Cr^6+^, As^3+^, and Pb^2+^ [19–24]. CD luminescent probes are less expensive, simple to synthesize, and have adjustable fluorescence. CDs are synthesized using plant extracts, carbon-based materials, animal byproducts, and synthetic compounds [25–29]. During their purification, CDs were first discovered from single-walled carbon nanotubes (CNTs) in 2004 [30]. Since then, much research has been conducted on them due to their excellent properties and applications [31]. CDs are used in optronics, biomedicine, catalysis, photodynamic therapy, and sensing due to their easy-to-tune properties with different heteroatoms and metals, small size, good fluorescence, and low toxicity [32–35].

Zhao et al. synthesized N and B-doped CDs via the hydrothermal method for the fluorescence sensing of Co^2+^ ions in biological and aqueous samples having a detection limit of 0.52 nM [36]. Dastidar et al. used onion extract to synthesize CDs via a hydrothermal route and employed it in sensing Zn^2+^ ions in blood plasma with a detection limit of 6.4 µM [37]. Deng et al. synthesized N-doped CDs from the biomass tar obtained from wood chips and showed its application in sensing Fe^3+^ ions by fluorescence quenching with a detection limit of 60 nmol/L [38]. Mu et al. used copper carbon dots synthesized from ascorbic acid and ethylenediaminetetraacetic acid disodium salt (Na_2_[Cu (EDTA)]) for the detection of Fe^3+^ ions and tryptophan with the detection limit of 46 nM and 275 nM, respectively [39]. Pimsin et al. used citric acid and dimethylglyoxime (DMG) to synthesize graphene quantum dots (GQDs) via the pyrolysis method, having a quantum yield of 49%. The synthesized material was used for the effective sensing of Ni^2+^, having a detection limit of 20.0 μg/L [40]. Zhao et al. used the purple perilla plant to synthesize CDs through the hydrothermal route and employed it in sensing Ag^+^ ions in aqueous samples with 1.4 nM as the detection limit [41]. Kayani et al. synthesized nitrogen-doped CDs as a fluorescence probe for sensing ascorbic acid and Fe(III) with detection limits of 0.69 µM and 290 µM, respectively [42]. Similarly, in another study, Kayani et al. used phosphorous-doped CDs to detect tetracyclines in tap water and milk samples assisted with smartphones [43].

Chromium (Cr) is a carcinogenic and hazardous heavy metal widely used in various sectors, including tannery, metallurgy, ceramics, cement, and pigment [44, 45]. The second most common atmospheric pollutant is hexavalent chromium [Cr(VI)] contamination. Even low concentrations of Cr(VI) can cause health risks such as kidney and liver damage, nasal septal perforation, nasal mucosa irritation, and ulcers [46, 47]. The US Environmental Protection Agency (EPA) and the World Health Organization (WHO) have recommended that the maximum allowable concentrations of Cr(VI) should be 100 µg/L and 50 µg/L, respectively [48, 49]. Because Cr(VI) is almost 50–100 times more hazardous than Cr(III), both its identification and remediation have received significant concern [50]. Liu et al. used N-CDs for sensing Cr(VI) ions by fluorescence quenching with a detection limit of 0.26 um [9]. Luo et al. used Cu-CDs synthesized from activated carbon derived from a *luffa sponge* for sensing Cr(VI) in water samples by fluorescence quenching [50]. The selectivity is a crucial sensing feature since many metal ions, including Pb^2+^, K^+^, Fe^3+^, Ca^2+^, Mn^2+^, Ni^2+^, Mg^2+^, Fe^2+^, Zn^2+^, Co^2+^, As^3+^, Cu^2+^, Cd^2+^, Pd^2+^, and Cr^3+^ compete with the target ions.

Many works have been reported on the sensing of Cr(VI) ions using CDs, but there is always a need for a selective sensor with low detection limits. In this article, we present the novel hydrothermal synthesis of copper-doped CDs (Cu-CDs) and their application in the sensing of chromium ions in aqueous samples. We used copper (Cu) as a dopant because of its advantages: low cost, enhanced stability, superior conductivity, bio-compatibility, and abundant availability. Moreover, the incorporation of copper in CDs enhances their donor and acceptor ability. This enhances the fluorescence properties of CDs and is also helpful in providing selectivity towards the sensing of ions. Also, the high stability of Cu-CDs showed that the synthesized material is applicable at different temperatures, pH, and light conditions. The as-synthesized Cu-CDs’ size, morphology, and surface functionalities were evaluated using numerous analytical techniques like HRTEM, UV–vis, Raman, FTIR, and XPS studies. The Cu-CDs showed superior fluorescent properties and were selective towards sensing Cr(VI) ions in aqueous samples by fluorescence quenching.

## Experimental section

### Materials

All the chemicals and reagents used like citric acid (C_6_H_8_O_7_), urea (CO(NH_2_)_2_), copper chloride (CuCl_2_), hydrochloric acid (HCl), sodium hydroxide (NaOH), sodium chloride (NaCl), palladium chloride (PdCl_2_), nickel chloride (NiCl_2_), ferrous chloride (FeCl_2_), magnesium chloride (MgCl_2_), potassium nitrate (KNO_3_), manganese chloride (MnCl_2_), cobalt chloride (CoCl_2_), zinc chloride (ZnCl_2_), calcium chloride (CaCl_2_), potassium dichromate (K_2_Cr_2_O_7_), cadmium chloride (CdCl_2_), lead nitrate (Pb(NO_3_)_2_), and arsenic oxide (As_2_O_3_) were of analytical grade and were purchased from Sigma-Aldrich (Merck) and SRL chemicals, India.

### Synthesis of Cu-CDs

**Fig. 1:**
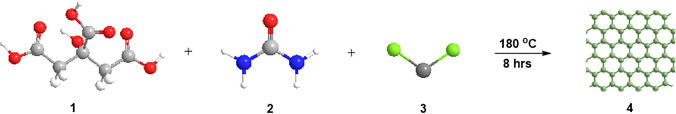
Synthesis of Cu-CDs through hydrothermal carbonization

The Cu-CDs were prepared using the method previously reported in our earlier research [51]. This work used citric acid **1** (0.01 M) instead of ascorbic acid as a carbon source. Then urea **2** (0.01 M) and cupric chloride **3** (0.015 M) were added as nitrogen and copper sources to the solution to synthesize Cu-CDs **4,** as depicted in Fig. 1. The samples were placed in a muffle furnace at 180 °C for 10 h. The resultant solution was dialyzed using a dialysis membrane (Cole-Parmer, MWCO = 1000) and centrifuged to eradicate impurities from the solution. As reported earlier, the same procedure was followed for filtration and lyophilization purposes, and the prepared samples were analyzed and employed in degradation studies [51]. Three distinct batches of Cu-CDs were produced and analyzed to guarantee repeatability, revealing slight variations in size and fluorescence intensity (± X%). Furthermore, we investigated scalability by augmenting reactant concentrations, verifying that the optical characteristics were consistent for samples produced at more significant volumes.

### Instrumentation

The particles’ surface morphology and particle size were analyzed using a high-resolution transmission electron microscopy (HR-TEM) (FP 5022/22-Tecnai G2 20 S-TWIN) microscope. The Fourier transform infrared spectrum (FTIR) was obtained using PerkinElmer Spectrum 2, from 400 to 4000 cm^−1^ with the KBr pellet technique. The X-ray photoelectron spectroscope (XPS) spectra were carried out in a (Nexsa base, Thermo-fisher scientific) X-ray photoelectron spectrophotometer. The Raman spectra were obtained in a (Lab RAM HR evolution, Horiba) Raman spectrophotometer. The UV–vis absorption spectra were measured on a (LT-2201) UV–vis spectrophotometer. The fluorescence studies were observed in a (Cary-Eclipse, Agilent) fluorescence spectrophotometer using a 150W CW Ozone-free Xenon arc lamp.

### Fluorescent studies

The as-synthesized Cu-CDs were dissolved in Millipore water to prepare a stock solution with a 0.1 mg/mL concentration. The prepared stock solution was then serially diluted to obtain a solution with Cu-CDs of 1 ug/mL for fluorescence analysis. The emission spectra were scanned using 320 nm as the excitation wavelength, with a slit width of 5 nm and a scanning speed of 120 nm/min in the range of 330–630 nm. To determine the effect of Cr(VI) ions on fluorescence intensity, 2.0 mL of a solution containing 1.0 ug/mL of Cu-CDs was added to a colorimetric tube. Then, 2.0 mL of a Cr(VI) ion solution with a concentration of 10 mM was added sequentially, making the final volume of the solution to 4.0 mL. The mixture was then shaken for 30 s and analyzed for fluorescence intensity at *λ*_ex_ = 340 nm and *λ*_em_ = 438 nm. Similarly, the fluorescence intensity for different metal ions was analyzed following the same procedure.

## Results and discussion

### Surface morphology

**Fig. 2:**
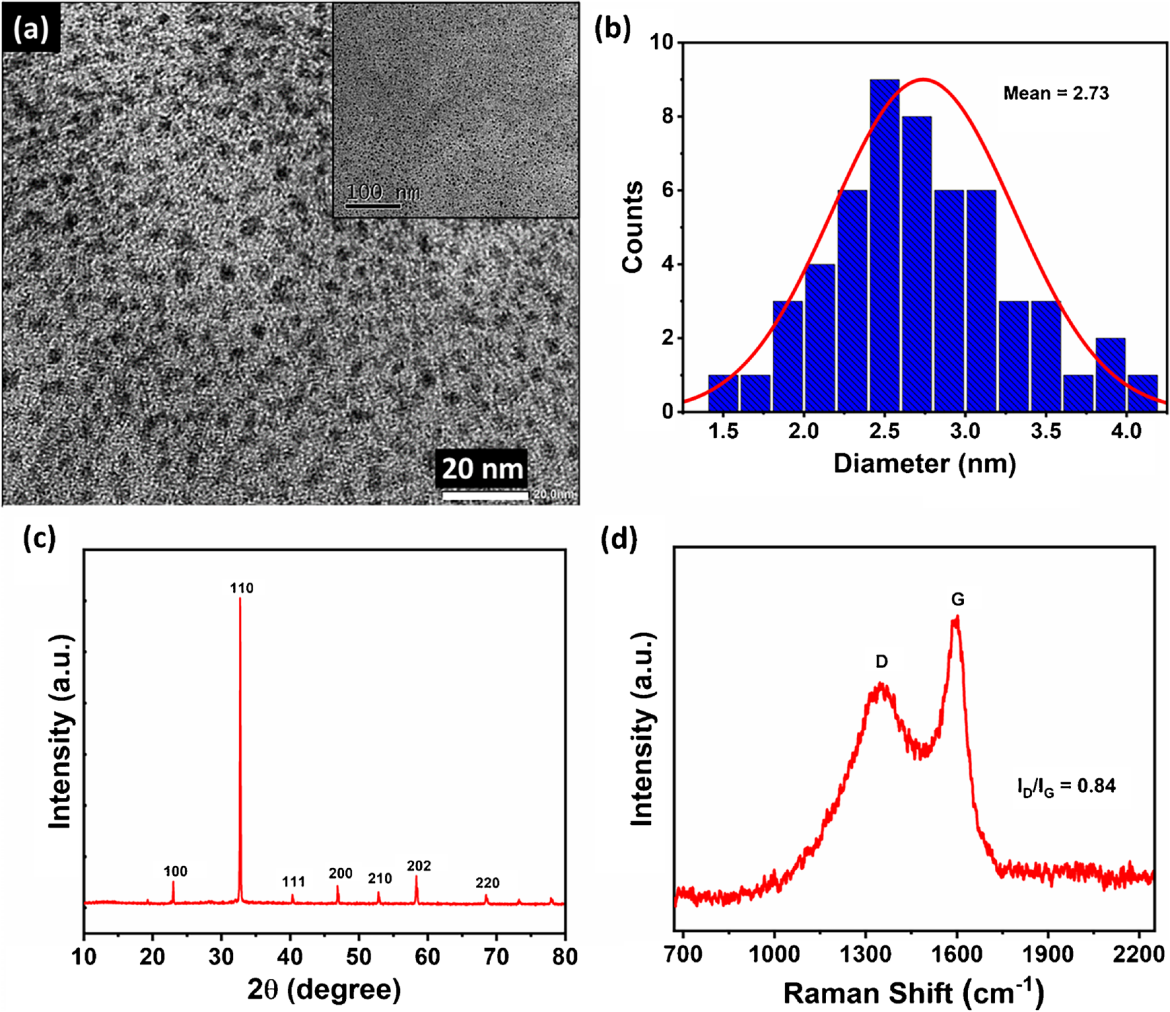
**a** HRTEM images, **b** particle size distribution, **c** XRD spectra, and **d** Raman spectrum of Cu-CDs

The structure and morphology of as-synthesized Cu-CDs were evaluated using high-resolution transmission electron microscopy (HRTEM). From the HRTEM images shown in Fig. 2a, it is evident that Cu-CDs are quasi-spherical in shape. The uniform dispersibility of Cu-CDs is evident from the inset of Fig. 2a. The Cu-CDs possess a particle diameter of 1.0 to 4.5 nm and an average particle size of 2.73 nm, as shown in Fig. 2b. The nature of the synthesized material was analyzed using the XRD spectrum as depicted in Fig. 2c. The peaks at 23.5° correspond to the (002) plane of graphitic carbon [51, 52]. The other prominent peak at 32.7° corresponds to the (110) plane of copper [53]. The other small peaks are also characteristic peaks of copper in different bonding types [54]. The Raman spectra of Cu-CDs (Fig. 2d) ascribe the D and G bands at 1350.20 and 1597.74 cm^−1^, corresponding to disordered C-atoms that are present at the edges of Cu-CDs due to surface functionalities and sp^2^-bonded C atoms, respectively [55, 56]. The intensity ratio calculated for D and G bands (*I*_D_/*I*_G_) is 0.84, which shows the high degree of graphitization of Cu-CDs.

### Surface functionalization

**Fig. 3:**
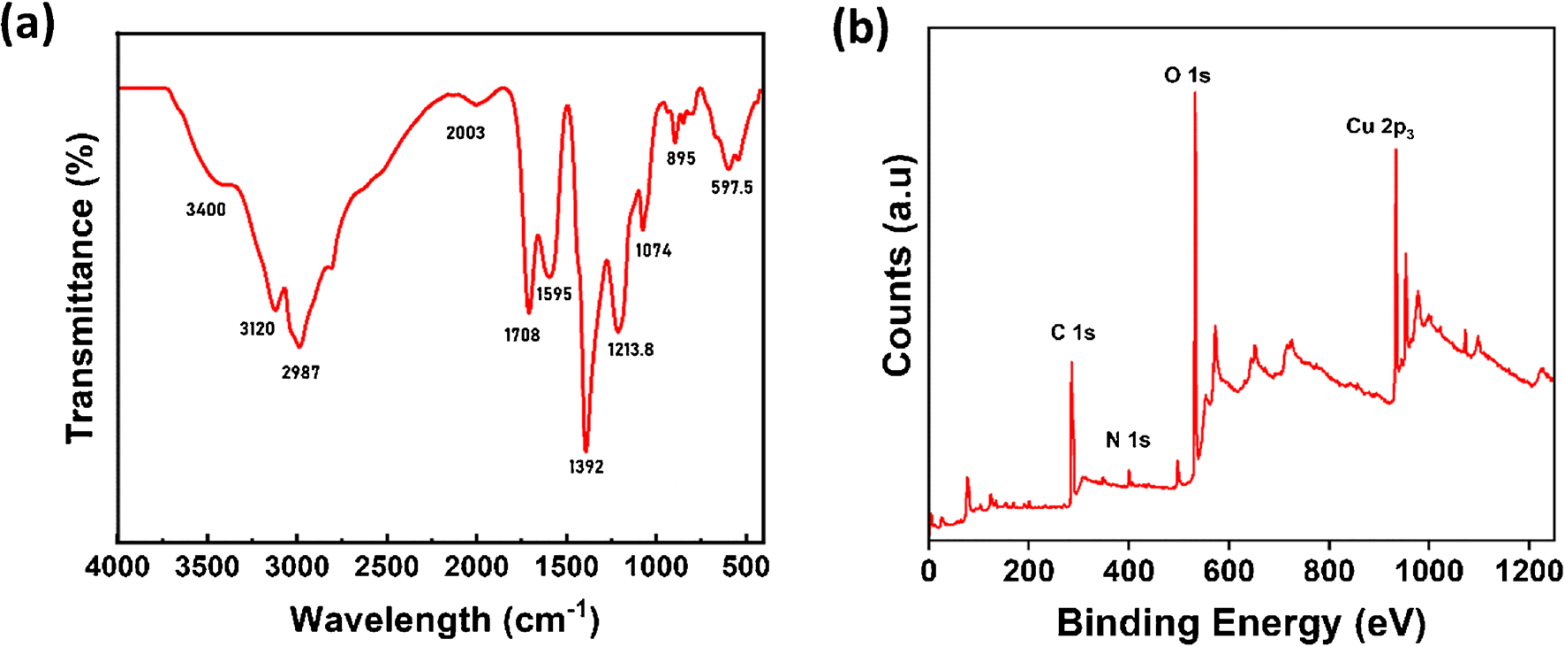
**a** FTIR spectrum and **b** XPS survey spectra of Cu-CDs

The FTIR spectra (Fig. 3a) show distinct absorption bands between 400 and 4000 cm^−1^. The peaks at 3400 cm^−1^ and 3120.3 cm^−1^ could be attributed to the stretching frequency of hydroxyl and N − H groups. The absorption bands at 2987 cm^−1^ and 2003 cm^−1^ can be ascribed to the stretching and bending frequency of C − H, respectively [51]. The bands obtained at 1708 cm^−1^ and 1595 cm^−1^ show the absorption band of C = O and C = C stretching vibrations [57]. The peaks at 1392 cm^−1^ and 1213.8 cm^−1^ could correspond to the bending and stretching frequency of O − H and C − N groups, respectively. The absorption bands between 800 and 1250 cm^−1^ can be ascribed to the stretching vibrations due to Cu − N and N − Cu − N groups in Cu-CDs [58].

**Fig. 4:**
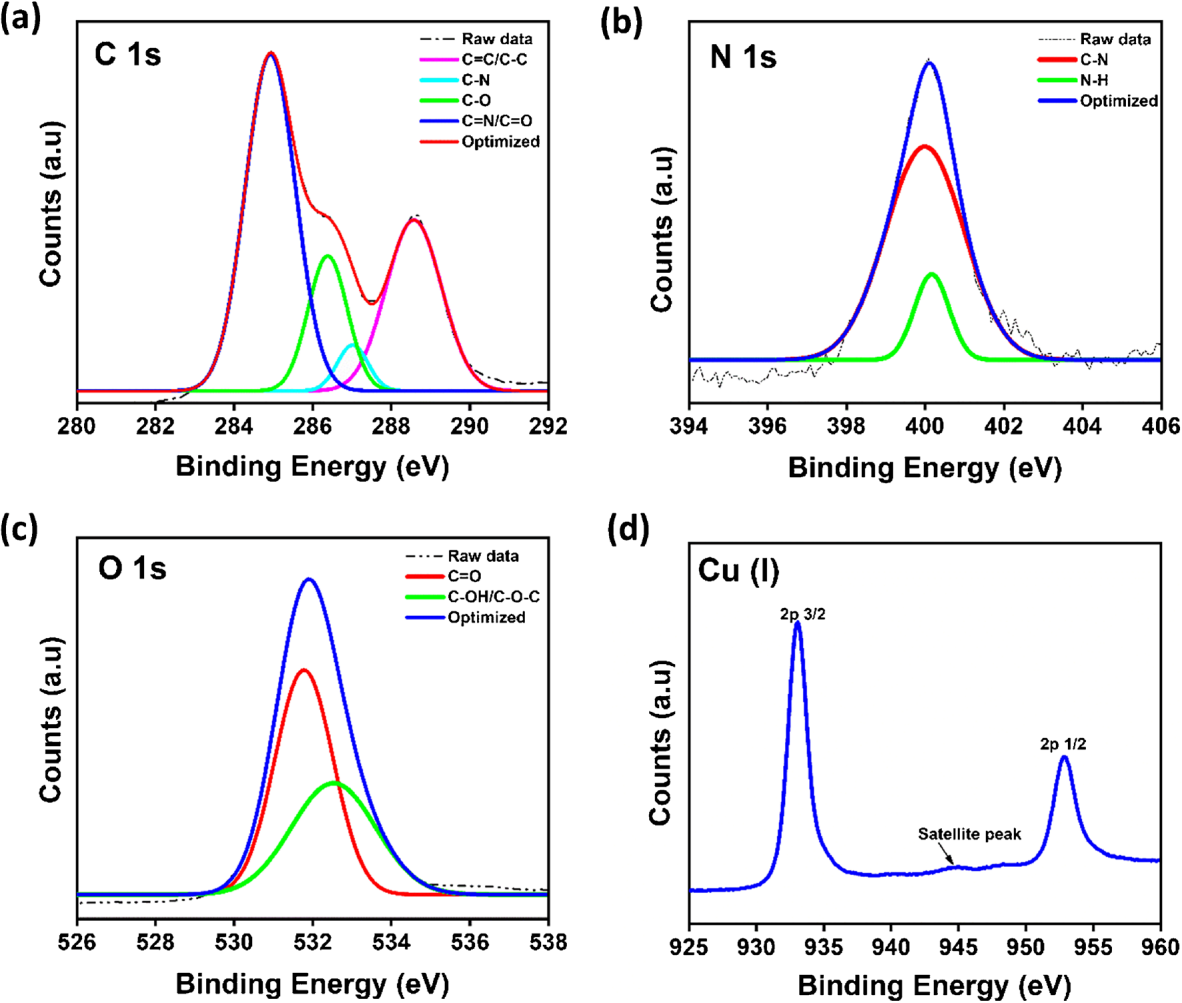
High-resolution XPS spectra of **a** carbon 1 s, **b** oxygen 1 s, **c** nitrogen 1 s, and **d** copper 2p of Cu-CDs

The elemental composition and surface functionalities of as-synthesized Cu-CDs were further confirmed by XPS analysis. The XPS survey spectra (Fig. 3b) of Cu-CDs confirm the existence of C, N, O, and Cu and their respective contents as 51.31%, 3.45%, 39.26%, and 5.99%. The high-resolution XPS spectrum of C 1 s (Fig. 4a) shows different bands at 284.8, 286.3, 286.9, and 288.5 eV, which are ascribed to C = N/C = O, C − O, C − N, and C = C/C − C groups, respectively. The O 1 s scan (Fig. 4b) shows two peaks at 531.7 and 532.5 eV, corresponding to C = O and C − OH groups. The two peaks in N 1 s spectra (Fig. 4c) at 399.9 and 400.2 eV are attributed to C − N and N − H groups. The Cu 2p spectrum in Fig. 4d exhibits two peaks at 933.1 and 952.8 eV, corresponding to Cu 2p_3/2_ and Cu 2p_1/2_, confirming the presence of Cu ions in Cu-CDs [58, 59]. The weak satellite peak around 944.5 eV confirms the presence of Cu(I) in the Cu-CDs [60]. The results show that the Cu-CDs synthesized are in the (+ 1) oxidation state.

### Optical properties

**Fig. 5:**
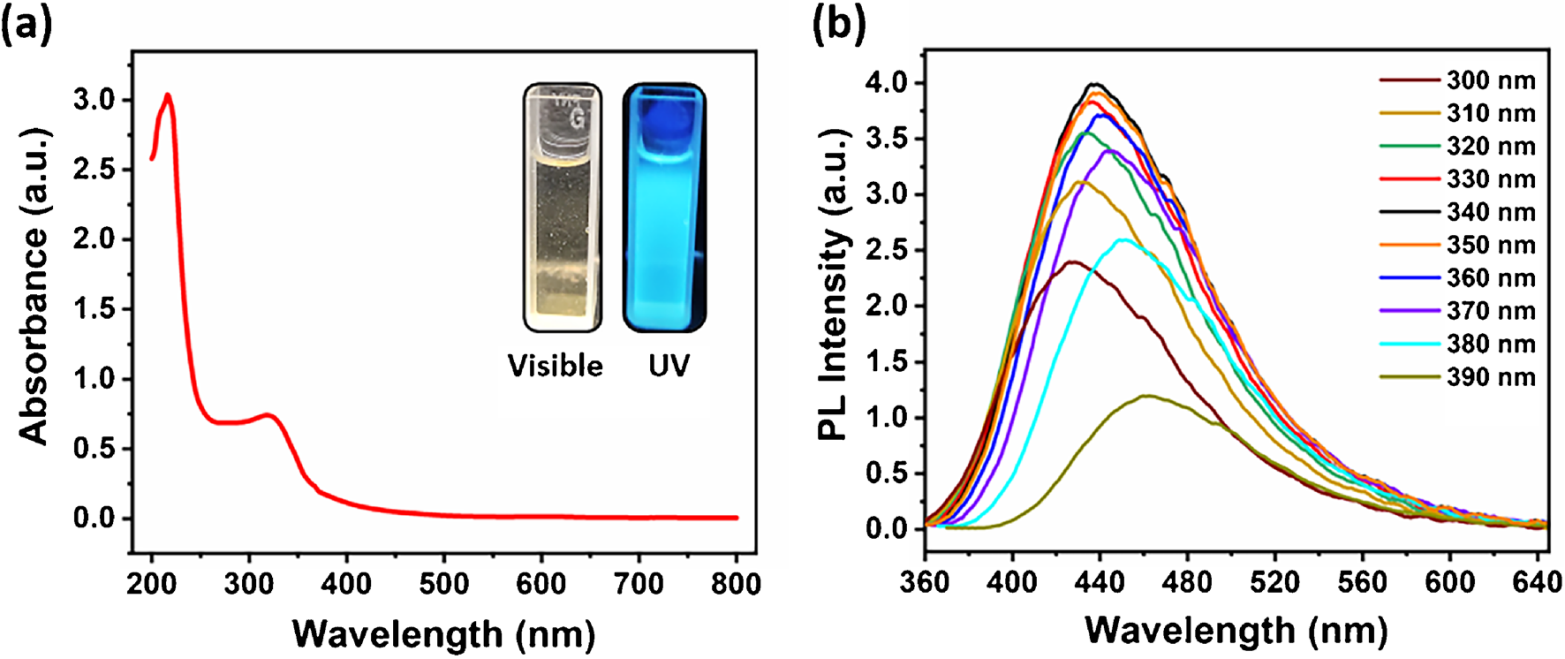
**a** UV–vis absorption spectrum (inset showing the picture of Cu-CDs under visible light and UV light at 365 nm) and **b** emission spectra of the Cu-CDs under different excitation wavelengths

The fluorescence and spectrophotometric characteristics of the produced Cu-CDs were studied by UV–vis adsorption and PL emission spectroscopy. The initial CDs solution was translucent yellow, but it generated blue solid fluorescence when exposed to a 365 nm portable UV lamp, as shown in Fig. 5a. In addition, Fig. 5a illustrates the UV–vis absorption spectra of Cu-CDs, which shows two absorption bands at 216 nm and 320 nm. The band at 216 nm is attributed to the π–π* transition corresponding to C = C groups. The absorption band at 320 nm is attributed to the n–π* transition corresponding to the nitrogen and oxygen-containing functionalities (C = O) of Cu-CDs. As synthesized, the photoluminescence spectra of pure Cu-CDs are shown in Fig. 5b at various excitation wavelengths ranging from 300 to 390 nm. The fluorescence emission spectra demonstrate that the most intense fluorescence band was observed at 438 nm after excitation at 340 nm. The fluorescence emission intensity steadily rose as the *λ*_ex_ increased from 300 to 340 nm, reaching a maximum at *λ*_ex_ = 340 nm. The further rise in *λ*_ex_ from 340 to 390 nm resulted in a gradual drop in fluorescence intensity. As λ_ex_ increased, the fluorescence emission spectra redshifted somewhat from 438 to 461 nm.

### Stability of Cu-CDs

**Fig. 6:**
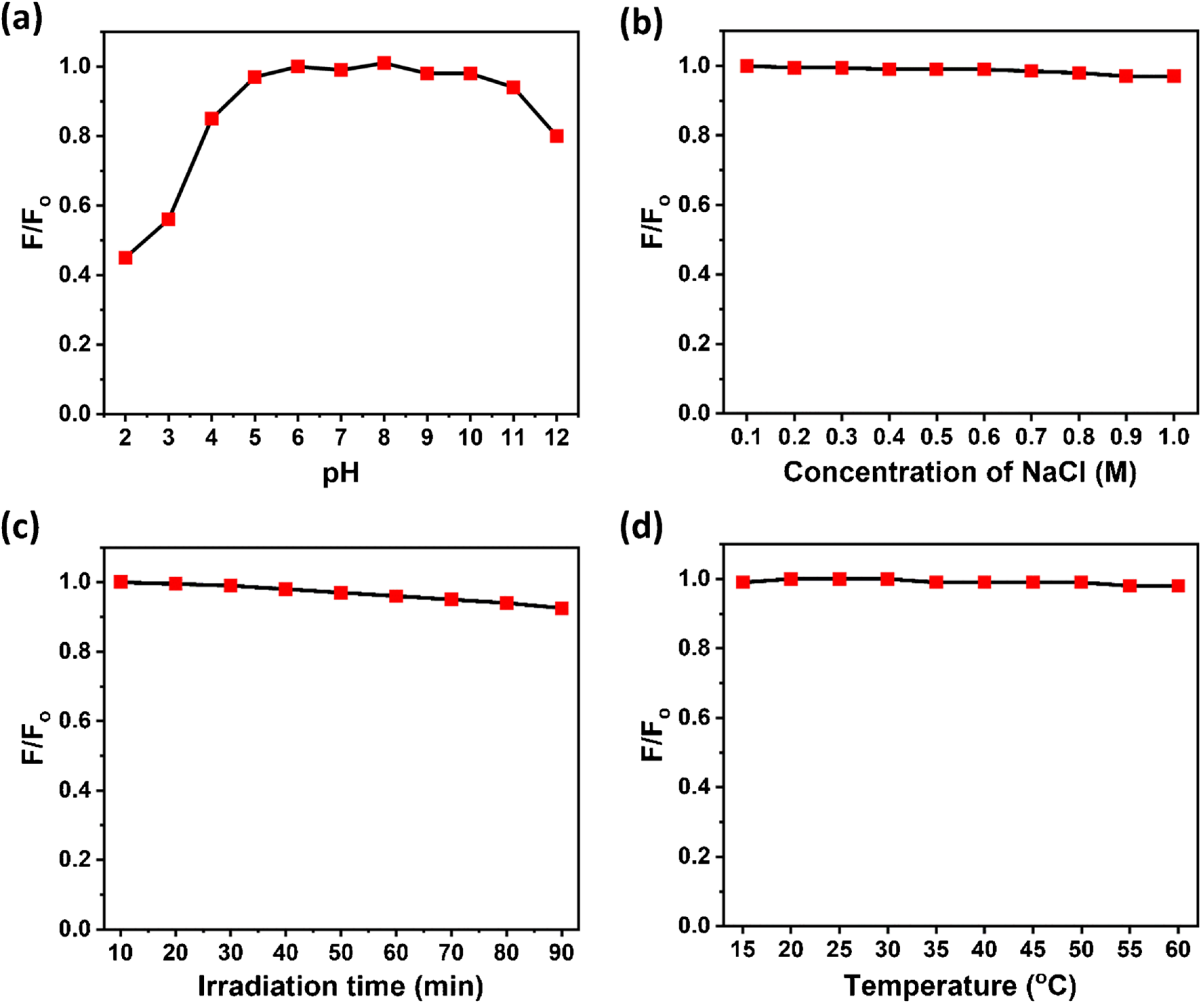
**a** Effect of pH, **b** ionic strength, **c** UV exposure, and **d** temperature on the stability of Cu-CDs

The photo and chemical stability of Cu-CDs is significant for their application in different sectors. The influence of pH on the stability of Cu-CDs was examined. Since pH might affect a sensor’s selectivity and sensitivity in aqueous systems. The fluorescence intensity of Cu-CDs varied only slightly across an extensive pH range (pH 4–10), but their fluorescence intensity altered significantly in both highly acidic (pH 2–4) and highly basic (pH 11–12) environments, as illustrated in Fig. 6a. This decreased intensity can be ascribed to more breakdown of the oxygen moieties that are dispersed arbitrarily on the surface of Cu-CDs and their associated fluorescent properties. Thus, the intensity of fluorescence for Cu-CDs is examined with different NaCl concentrations to assess the ionic strength resistance of Cu-CDs as shown in Fig. 6b. The results show minimal change in fluorescence intensity after the NaCl concentration approaches 1.0 M, which demonstrates that the Cu-CDs are resistant to salt. The stability of Cu-CDs was also investigated under UV light irradiation for 10 to 90 min. The Cu-CDs showed more excellent stability under UV light, and a very low change in fluorescence intensity was observed even after 90 min, as depicted in Fig. 6c. The influence of temperature on the stability of Cu-CDs was studied. The Cu-CDs showed excellent stability in the range of 15 to 60 °C, and no change in fluorescence intensity was observed, as depicted in Fig. 6d. The stability of Cu-CDs was also investigated after being stored for 180 days. The Cu-CDs exhibited excellent fluorescence after 180 days of storage under ordinary light. Also, the quantum yield (QY) of the Cu-CDs was found to be 27.3% and was calculated using quinine sulfate as reference material using the relation (1)

1 $$QY\left(\phi \right)=\frac{{I}_{CDs }{ ({\eta }_{ref})}^{2}}{{I}_{ref }{({\eta }_{CDs})}^{2}}.{\phi }_{ref}$$

where *Φ*_ref_ is the quantum yield, *I*_ref_ is the intensity of the emitted light, and η_ref_ is the refractive index of the solvent for quinine sulfate. *I*_CDs_ is the intensity of the emitted light and η_CDs_ is the refractive index of the solvent used for the carbon dots.

### Sensing of Cr(VI) ions

**Fig. 7:**
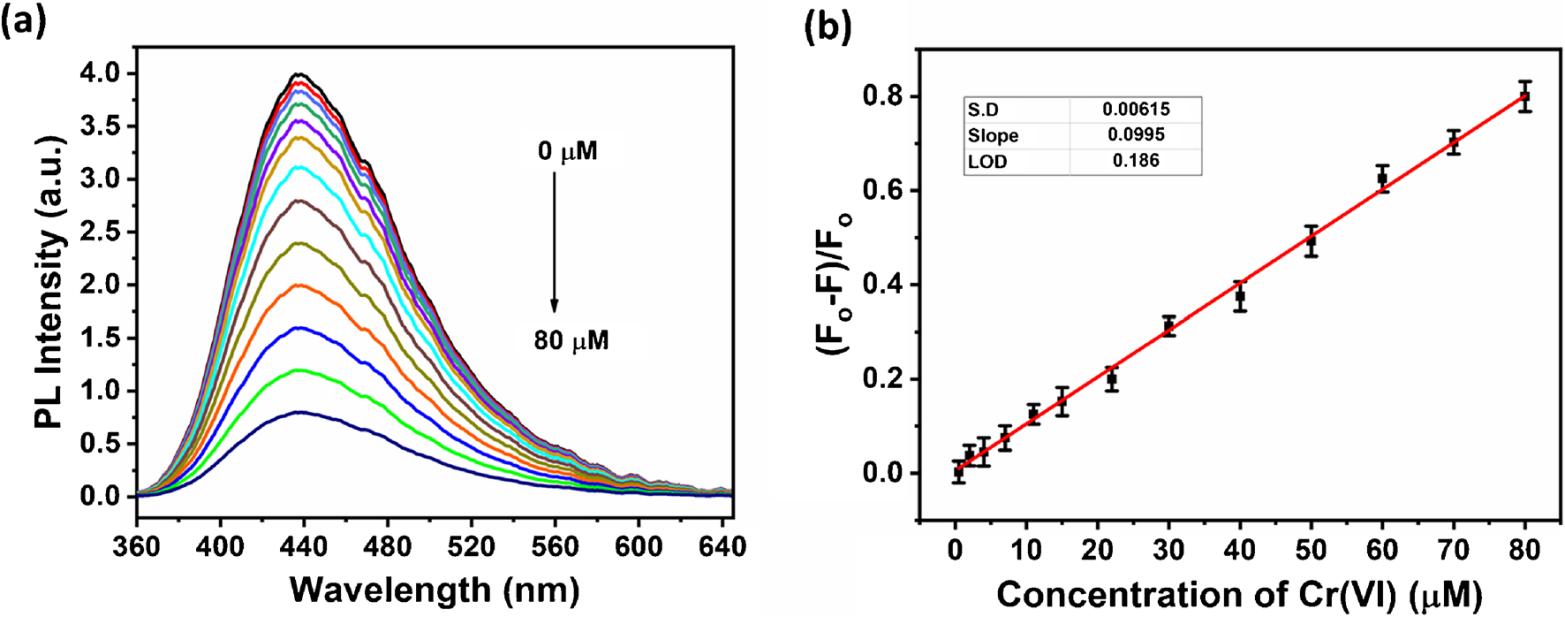
**a** Emission spectra of Cu-CDs with increasing Cr(VI) concentration (0–80 µM) and **b** relationship between (*F*_0_-*F*)/*F*_0_ and concentration of Cr(VI) ions at *λ*_ex_ = 340 nm

The influence of Cr(VI) ions on the PL intensity of the Cu-CDs was investigated to assess the applicability of the synthesized Cu-CDs as a fluorescence probe for Cr(VI). The emission bands observed at various Cr(VI) concentrations are shown in Fig. 7a. The luminescence intensity of the Cu-CDs steadily decreased at 438 nm emission as the concentration of Cr(VI) increased from 0 to 80 µM, indicating that Cr(VI) has a quenching effect on the luminescence of the Cu-CDs. The fluorescence intensity of the Cu-CDs and the concentration of Cr(VI) are well correlated, as shown by the acquired linear correlation coefficient *R*^2^ of 0.989, as illustrated in Fig. 7b. *F*_0_ is the luminescence intensity of the Cu-CDs at 438 nm emission when Cr(VI) is not added. At the same time, *F* is the luminescence intensity of the Cu-CDs at 438 nm emission when Cr(VI) is added in various quantities. The outcomes demonstrate the Cu-CDs’ bright future as a quick and straightforward method for determining Cr(VI). The Limit of detection (LOD) and limit of quantification (LOQ) were determined using the formulas LOD = 3 *σ*/*K*_SV_ and LOQ = 10 *σ*/*K*_SV_, respectively, where *σ* is the standard error of the intercept from the Stern–Volmer plots [18]. Cr(VI) had a LOD of 0.186 µM, which is much lower than the permissible limits by WHO (0.96 µM) [9] and a LOQ of 0.618 µM, where *σ* = 0.00615 and *K*_SV_ = 0.0995 with a *R*^2^ value of 0.997. The emission intensities for the pure Cu-CDs and [Cu-CDs-Cr(VI)] solutions at 438 nm, demonstrating a minute variation over time when the samples were subjected to continuous irradiation at 340 nm for 900 s, further showed the usefulness of Cu-CDs as a sensor for Cr(VI) ions.

**Fig. 8:**
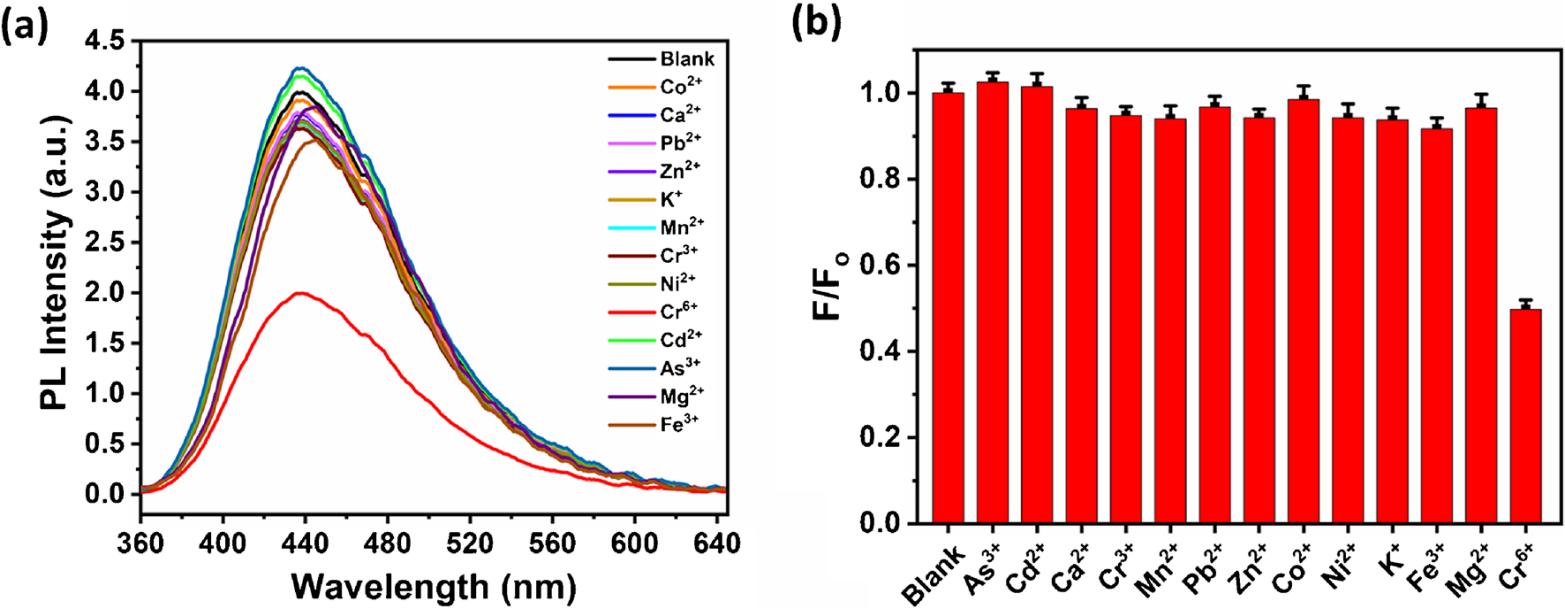
**a** Emission spectra of Cu-CDs in the presence of different metal ions and **b** fluorescence response (*F*/*F*_0_) of Cu-CDs for different metal ions at *λ*_ex_ = 340 nm

Additionally, the impact of various metal ions on the selectivity coefficient (*F*/*F*_0_) for fluorescence quenching of Cu-CDs was examined by introducing different metal ions (at a concentration of 50 µM) such as K^+^, Mg^2+^, Ca^2+^, Mn^2+^, Fe^3+^, As^2+^, Co^2+^, Ni^2+^, Pb^2+^, Zn^2+^, Cd^2+^, Cr^6+^, and Fe^3+^ to a Cu-CD solution. Only Cr(VI) considerably reduces the fluorescence emission of the Cu-CDs, as shown in Fig. 8a, demonstrating the Cu-CDs’ strong selectivity for Cr(VI) detection in aqueous solution. The fluorescence quenching effect may be observed by introducing Cr(VI) in the presence of potential interference metal ions. As seen in Fig. 8b, the *F*/*F*_0_ value fluctuates very little for different metal ions, and only a decrease in fluorescence intensity value was observed for Cr(VI) ions, demonstrating the Cu-CDs’ strong anti-interference capacity for Cr(VI) ion detection. Also, the effect of the mixture of metal ions with Cr(VI) on the fluorescence intensity has been investigated. It was observed that all the metal ions (K^+^, Mg^2+^, Ca^2+^, Mn^2+^, Fe^3+^, As^2+^, Co^2+^, Ni^2+^, Pb^2+^, Zn^2+^, Cd^2+^, Cr^6+^, and Fe^3+^) has a very minute effect on the quenched fluorescence intensity. This showed that sensing Cr(VI) has no interfering effect by the presence of other metal ions.

Fluorescence titration studies were conducted to demonstrate the specifics of the sensing mechanism. The drop in intensity indicated the interaction of the Cu-CDs and Cr(VI) ion. At the same time, the quenching of the Cu-CDs’ bright blue color might result from the static quenching, inner filter effect, dynamic quenching, or a mixture of any two. As reported in previous literature, the absorption spectra of Cr(VI) exhibit three peaks at 438 nm, 351 nm, and 258 nm. Still, the Cu-CDs show absorption bands at 216 nm and 320 nm and an emission band at 438 nm at an excitation wavelength of 340 nm.

**Fig. 9:**
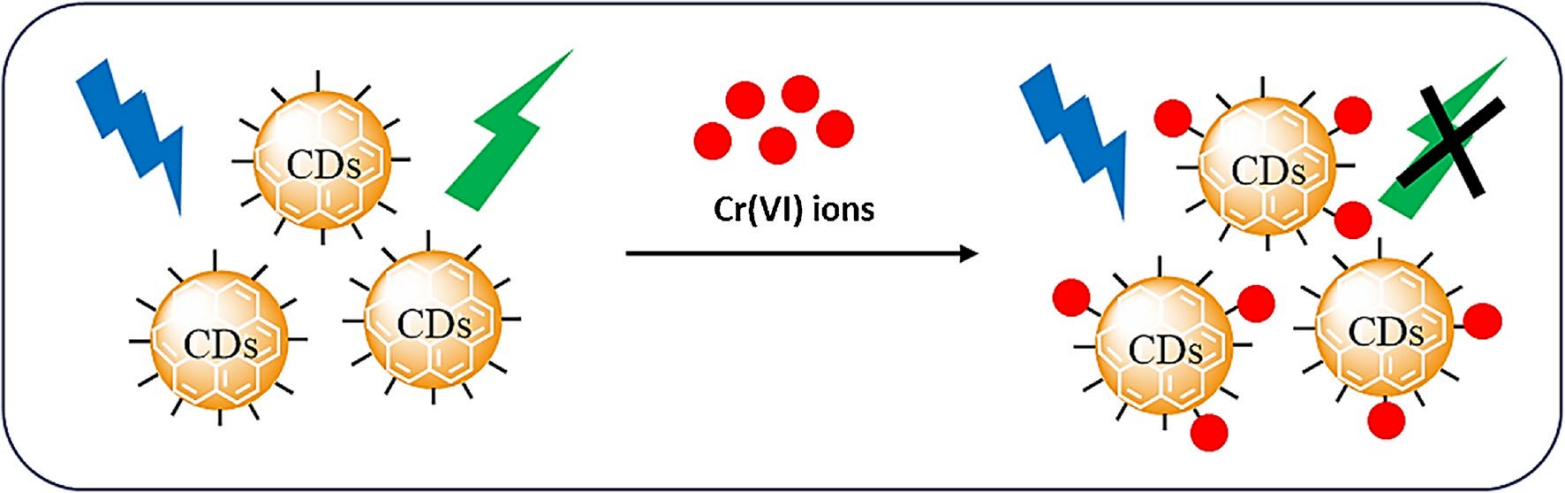
Fluorescence quenching mechanism for Cu-CDs by Cr(VI) ions

As a consequence, the excellent spectrum overlapping of absorber Cr(VI) and fluorophore (Cu-CDs) results in the sheltering of Cu-CD emission and excitation bands by absorber Cr(VI), resulting in fluorescence quenching. The Cr(VI) ions attach to the fluorophores on the surface of Cu-CDs, which decreases fluorescence, as depicted in Fig. 9. This shows that Cu-CDs have good selectivity and sensitivity for Cr(VI) ions. The synthesized material can be further applied in sensing Cr(VI) ions in real water samples. It can be applicable in detecting ions above their limits by regulating their concentrations in water samples. Also, the detection limit of Cu-CDs is much lower than the permissible limits for drinking water by WHO, and a comparison of other catalysts for removing Cr(VI) ions is also listed in Table 1. Preliminary analyses were performed on actual water samples obtained from industrial effluent locations. Cr(VI) concentrations were introduced into these samples, and Cu-CD-based sensors were used. The observed fluorescence quenching aligned with the laboratory findings, suggesting that this method efficiently detects Cr(VI) in real-world applications.

**Table 1:** Comparison of other catalysts for the sensing of Cr ions

S. no	Catalyst used	Ion sensing	LOD (µM)	Linear range (µM)	Ref
1	MOF + CDs	Cr(VI)	0.16	0–200	[61]
2	Amino—CDs	Cr(VI)	0.14	0.25–25	[62]
3	Dual-emission carbon dots (DECD)	Cr(VI)	0.14	2–300	[63]
4	Sulfur QDs	Cr(VI)	0.36	20–500	[64]
5	Porous CDs	Cr(VI)	1.491	0–2.5	[65]
6	Cu-CDs	Cr(VI)	0.185	0–80	This study

## Conclusion

In this article, we have discussed the novel synthesis of copper-doped carbon dots (Cu-CDs) from citric acid as a carbon source, urea as a nitrogen source, and CuCl_2_ as a copper source through the hydrothermal route. The prepared materials’ size, morphology, and surface functionalities were confirmed using analytical techniques like HRTEM, UV–vis, Raman, FTIR, and XPS studies. The fluorescent properties of the Cu-CDs were analyzed using fluorescence and UV–vis spectrometer. The Cu-CDs showed excellent optical stability and dispersibility in water and exhibited blue fluorescence. Furthermore, the Cu-CDs showed a significant emission band at 438 nm (*λ*_em_) when excited at 340 nm (*λ*_ex_). Notably, the Cu-CDs maintained fluorescence stability across a wide pH range (4–11) (Fig. 6a), while demonstrating significant resistance to ionic interference (0.1 to 1.0 M NaCl) (Fig. 6b). The application of Cu-CDs for detecting metal ions in aqueous solutions was investigated using different metal ions. Among the different metal ions used, the Cu-CDs showed good selectivity towards sensing Cr(VI) ions due to the decrease in fluorescence, which may be due to the static quenching, inner filter effect, or dynamic quenching. A strong linear relationship in the 0–80 µM range has been observed in the sensing system for Cr(VI) ions with the LOD as low as 0.185 µM and LOQ of 0.618 µM. This is much lower than the various previously reported articles. This showed that the Cu-CD sensing system can be applied for sensing Cr(VI) ions in water systems.

## Data Availability

No datasets were generated or analysed during the current study.

## References

[1] Rathi BS, Kumar PS, Show PL (2021) A review on effective removal of emerging contaminants from aquatic systems: current trends and scope for further research. J Hazard Mater 409:124413. 10.1016/j.jhazmat.2020.124413 33183841 10.1016/j.jhazmat.2020.124413

[2] Lu Y, Liang X, Niyungeko C, Zhou J, Xu J, Tian G (2018) A review of the identification and detection of heavy metal ions in the environment by voltammetry. Talanta 178:324–338. 10.1016/j.talanta.2017.08.033 29136830 10.1016/j.talanta.2017.08.033

[3] Elbeltagi A, Aslam MR, Malik A, Mehdinejadiani B, Srivastava A, Bhatia AS, Deng J (2020) The impact of climate changes on the water footprint of wheat and maize production in the Nile Delta, Egypt. Sci Total Environ 743:140770. 10.1016/j.scitotenv.2020.140770 32679501 10.1016/j.scitotenv.2020.140770

[4] Singh R, Singh S, Kumar M (2020) Impact of n-butanol as an additive with eucalyptus biodiesel-diesel blends on the performance and emission parameters of the diesel engine. Fuel 277:118178. 10.1016/j.fuel.2020.118178

[5] Zamora-Ledezma C, Negrete-Bolagay D, Figueroa F, Zamora-Ledezma E, Ni M, Alexis F, Guerrero VH (2021) Heavy metal water pollution: a fresh look about hazards, novel and conventional remediation methods. Environ Technol Innov 22:101504. 10.1016/j.eti.2021.101504

[6] Shakoor MB, Ali S, Rizwan M, Abbas F, Bibi I, Riaz M, Khalil U, Niazi NK, Rinklebe J (2020) A review of biochar-based sorbents for separation of heavy metals from water. Int J Phytoremediation 22:111–126. 10.1080/15226514.2019.1647405 31686525 10.1080/15226514.2019.1647405

[7] Qian J, Cao N, Zhang J, Hou J, Chen Q, Zhang C, Sunb Y, Liub S, Heb L, Zhang K, Zhou H (2020) Field-portable ratiometric fluorescence imaging of dual-color label-free carbon dots for uranyl ions detection with cellphone-based optical platform. Chin Chem Lett 31:2925–2928. 10.1016/j.cclet.2020.05.004

[8] Malik LA, Bashir A, Qureashi A, Pandith AH (2019) Detection and removal of heavy metal ions: a review. Environ Chem Lett 17:1495–1521. 10.1007/s10311-019-00891-z

[9] Liu S, Cui J, Huang J, Tian B, Jia F, Wang Z (2019) Facile one-pot synthesis of highly fluorescent nitrogen-doped carbon dots by mild hydrothermal method and their applications in detection of Cr(VI) ions, Spectrochim. Acta Part A Mol Biomol Spectrosc 206:65–71. 10.1016/j.saa.2018.07.082 10.1016/j.saa.2018.07.082 30081269

[10] Li Q, Yang D, Yang Y (2021) Spectrofluorimetric determination of Cr(VI) and Cr(III) by quenching effect of Cr(III) based on the Cu-CDs with peroxidase-mimicking activity, Spectrochim. Acta Part A Mol Biomol Spectrosc 244:118882. 10.1016/j.saa.2020.118882 10.1016/j.saa.2020.118882 32919158

[11] Guo Y, Chen Y, Cao F, Wang L, Wang Z, Leng Y (2017) Hydrothermal synthesis of nitrogen and boron doped carbon quantum dots with yellow-green emission for sensing Cr(VI), anti-counterfeiting and cell imaging. RSC Adv 7:48386–48393. 10.1039/c7ra09785a

[12] Song Y, Tao J, Wang Y, Cai Z, Fang X, Wang S, Xu H (2021) A novel dual-responsive fluorescent probe for the detection of copper(II) and nickel(II) based on BODIPY derivatives. Inorganica Chim Acta 516:120099. 10.1016/j.ica.2020.120099

[13] Senol AM, Bozkurt E (2020) Facile green and one-pot synthesis of seville orange derived carbon dots as a fluorescent sensor for Fe^3+^ ions. Microchem J 159:105357. 10.1016/j.microc.2020.105357

[14] Kayani KF, Shatery OB, Mustafa MS, Alshatteri AH, Mohammed SJ, Aziz SB (2024) Environmentally sustainable synthesis of whey-based carbon dots for ferric ion detection in human serum and water samples: evaluating the greenness of the method. RSC Adv 14:5012–5021. 10.1039/D3RA08680A 38332781 10.1039/d3ra08680a PMC10851185

[15] Wang X, Feng Y, Dong P, Huang J (2019) A mini review on carbon quantum dots: preparation, properties, and electrocatalytic application. Front Chem 7:1–9. 10.3389/fchem.2019.00671 31637234 10.3389/fchem.2019.00671 PMC6787169

[16] Guo J, Lu W, Zhang H, Meng Y, Du F, Shuang S, Dong C (2021) Copper doped carbon dots as the multi-functional fluorescent sensing platform for tetracyclines and pH. Sensors Actuators, B Chem 330:129360. 10.1016/j.snb.2020.129360

[17] Zhang HC, Guo YM (2021) Advances of carbon quantum dots for fluorescence turn-on detection of reductive small biomolecules, Chinese. J Anal Chem 49:14–23. 10.1016/S1872-2040(20)60070-6

[18] Gao X, Du C, Zhuang Z, Chen W (2016) Carbon quantum dot-based nanoprobes for metal ion detection. J Mater Chem C 4:6927–6945. 10.1039/c6tc02055k

[19] Baragau IA, Power NP, Morgan DJ, Lobo RA, Roberts CS, Titirici MM, Middelkoop V, Diaz A, Dunn S, Kellici S (2021) Efficient continuous hydrothermal flow synthesis of carbon quantum dots from a targeted biomass precursor for on-off metal ions nanosensing. ACS Sustain Chem Eng 9:2559–2569. 10.1021/acssuschemeng.0c08594

[20] Kayani KF, Mohammed SJ, Ghafoor D, Rahim MK, Ahmed HR (2024) Carbon dot as fluorescence sensor for glutathione in human serum samples: a review. Mater Adv. 10.1039/D4MA00185K

[21] Ren Z, Wang J, Xue C, Deng M, Li Z, Zhang H, Cai C, Xu B, Wang X, Li J (2023) Carbon dot-functionalized solution-gated graphene transistors for highly sensitive detection of cobalt(II) ions. Chemosensors 11:1–11. 10.3390/chemosensors11030192

[22] Msto RK, Othman HO, Al-Hashimi BR, Salahuddin Ali D, Hassan DH, Hassan AQ, Smaoui S (2023) Fluorescence turns on-off-on sensing of ferric ion and L-ascorbic acid by carbon quantum dots. J Food Qual. 2023. 10.1155/2023/5555608.

[23] Yan Z, Yao W, Mai K, Huang J, Wan Y, Huang L, Cai B, Liu Y (2022) A highly selective and sensitive “on-off” fluorescent probe for detecting cadmium ions and l-cysteine based on nitrogen and boron co-doped carbon quantum dots. RSC Adv 12:8202–8210. 10.1039/d1ra08219a 35424768 10.1039/d1ra08219a PMC8982326

[24] Liu M, Li T, Zhang C, Zheng Y, Wu C, Zhang J, Zhang K, Zhang Z (2021) Fluorescent carbon dots embedded in mesoporous silica nanospheres: a simple platform for Cr (VI) detection in environmental water. J Hazard Mater 415:125699. 10.1016/j.jhazmat.2021.125699 33773242 10.1016/j.jhazmat.2021.125699

[25] Das GS, Shim JP, Bhatnagar A, Tripathi KM, Kim TY (2019) Biomass-derived carbon quantum dots for visible-light-induced photocatalysis and label-free detection of Fe(III) and ascorbic acid. Sci Rep 9:1–9. 10.1038/s41598-019-49266-y 31636279 10.1038/s41598-019-49266-y PMC6803716

[26] Liu F, Li H, Liao D, Xu Y, Yu M, Deng S, Zhang G, Xiao T, Long J, Zhang H, Li Y, Li K, Zhang P (2020) Carbon quantum dots derived from the extracellular polymeric substance of anaerobic ammonium oxidation granular sludge for detection of trace Mn(VII) and Cr(VI). RSC Adv 10:32249–32258. 10.1039/d0ra06133f 35518178 10.1039/d0ra06133f PMC9056554

[27] Kayani KF, Rahim MK, Mohammed SJ, Ahmed HR, Mustafa MS, Aziz SB (2024) Recent progress in folic acid detection based on fluorescent carbon dots as sensors: a review. J fluoresce. 1–14. 10.1007/s10895-024-03728-3. 10.1007/s10895-024-03728-3 38625574

[28] Prekodravac J, Vasiljević B, Marković Z, Jovanović D, Kleut D, Špitalský Z, Mičušik M, Danko M, Bajuk-Bogdanović D, Todorović-Marković B (2019) Green and facile microwave assisted synthesis of (metal-free) N-doped carbon quantum dots for catalytic applications. Ceram Int 45:17006–17013. 10.1016/j.ceramint.2019.05.250

[29] Shen TY, Jia PY, Chen DS, Wang LN (2021) Hydrothermal synthesis of N-doped carbon quantum dots and their application in ion-detection and cell-imaging, Spectrochim. Acta Part A Mol Biomol Spectrosc 248:119282. 10.1016/j.saa.2020.119282 10.1016/j.saa.2020.119282 33316652

[30] Xu X, Ray R, Gu Y, Ploehn HJ, Gearheart L, Raker K, Scrivens WA (2004) Electrophoretic analysis and purification of fluorescent single-walled carbon nanotube fragments. J Am Chem Soc 126:12736–12737. 10.1021/ja040082h 15469243 10.1021/ja040082h

[31] Zainal Abidin NH, Wongso V, Hui KC, Cho K, Sambudi NS, Ang WL, Saad B (2020) The effect of functionalization on rice-husks derived carbon quantum dots properties and cadmium removal. J Water Process Eng 38:101634. 10.1016/j.jwpe.2020.101634

[32] Nazri NAA, Azeman NH, Luo Y, Bakar AAA (2021) Carbon quantum dots for optical sensor applications: a review. Opt Laser Technol 139:106928. 10.1016/j.optlastec.2021.106928

[33] Rani UA, Ng LY, Ng CY, Mahmoudi E (2020) A review of carbon quantum dots and their applications in wastewater treatment. Adv Colloid Interface Sci 278:102124. 10.1016/j.cis.2020.102124 32142942 10.1016/j.cis.2020.102124

[34] He P, Shi Y, Meng T, Yuan T, Li Y, Li X, Zhang Y, Fan L, Yang S (2020) Recent advances in white light-emitting diodes of carbon quantum dots. Nanoscale 12:4826–4832. 10.1039/c9nr10958g 32065190 10.1039/c9nr10958g

[35] Kayani KF, Ghafoor D, Mohammed SJ, Shatery OB (2024) Carbon dots: synthesis, sensing mechanisms, and potential applications as promising materials for glucose sensors. Nanoscale Adv. 10.1039/D4NA00763H 39583130 10.1039/d4na00763h PMC11583430

[36] Zhao X, Wang L, Liu Q, Chen M, Chen X (2021) Facile synthesis of B, N-doped CQDs as versatile fluorescence probes for sensitive detection of cobalt ions in environmental water and biological samples. Microchem J 163:105888. 10.1016/j.microc.2020.105888

[37] Ghosh Dastidar D, Mukherjee P, Ghosh D, Banerjee D (2021) Carbon quantum dots prepared from onion extract as fluorescence turn-on probes for selective estimation of Zn^2+^ in blood plasma. Colloids Surfaces A Physicochem Eng Asp 611:125781. 10.1016/j.colsurfa.2020.125781

[38] Deng X, Feng Y, Li H, Du Z, Teng Q, Wang H (2018) N-doped carbon quantum dots as fluorescent probes for highly selective and sensitive detection of Fe^3+^ ions. Particuology 41:94–100. 10.1016/j.partic.2017.12.009

[39] Mu Z, Hua J, Feng S, Yang Y (2019) A ratiometric fluorescence and light scattering sensing platform based on Cu-doped carbon dots for tryptophan and Fe ( III ), Spectrochim. Acta Part A Mol Biomol Spectrosc 219:248–256. 10.1016/j.saa.2019.04.065 10.1016/j.saa.2019.04.065 31048254

[40] Pimsin N, Kongsanan N, Keawprom C, Sricharoen P, Nuengmatcha P, Oh WC, Areerob Y, Chanthai S, Limchoowong N (2021) Ultratrace detection of nickel(II) ions in water samples using dimethylglyoxime-doped GQDs as the induced metal complex nanoparticles by a resonance light scattering sensor. ACS Omega 6:14796–14805. 10.1021/acsomega.1c00190 34151061 10.1021/acsomega.1c00190 PMC8209797

[41] Zhao X, Liao S, Wang L, Liu Q, Chen X (2019) Facile green and one-pot synthesis of purple perilla derived carbon quantum dot as a fluorescent sensor for silver ion. Talanta 201:1–8. 10.1016/j.talanta.2019.03.095 31122398 10.1016/j.talanta.2019.03.095

[42] K.F. Kayani, C.N. Abdullah (2024) A dual-mode detection sensor based on nitrogen-doped carbon dots for visual detection of Fe (III) and ascorbic acid via a smartphone. J Fluoresc. 1–13. 10.1007/s10895-024-03604-0. 10.1007/s10895-024-03604-0 38300485

[43] Kayani KF, Mohammed SJ, Mohammad NN, Abdullah GH, Kader DA, Mustafa NSH (2024) Ratiometric fluorescence detection of tetracycline in milk and tap water with smartphone assistance for visual pH sensing using innovative dual-emissive phosphorus-doped carbon dots. Food Control. 110611. 10.1016/j.foodcont.2024.110611.

[44] Mohanty S, Benya A, Hota S, Kumar MS, Singh S (2023) Eco-toxicity of hexavalent chromium and its adverse impact on environment and human health in Sukinda Valley of India: a review on pollution and prevention strategies. Environ Chem Ecotoxicol 5:46–54. 10.1016/j.enceco.2023.01.002

[45] Garcia-Miranda Ferrari A, Crapnell RD, Adarakatti PS, Suma BP, Banks CE (2022) Electroanalytical overview: the detection of chromium. Sensors and Actuators Reports 4:100116. 10.1016/j.snr.2022.100116

[46] Sakaew C, Sricharoen P, Limchoowong N, Nuengmatcha P, Kukusamude C, Kongsri S, Chanthai S (2020) Green and facile synthesis of water-soluble carbon dots from ethanolic shallot extract for chromium ion sensing in milk, fruit juices, and wastewater samples. RSC Adv 10:20638–20645. 10.1039/d0ra03101a 35517751 10.1039/d0ra03101a PMC9054292

[47] Irshad MA, Sattar S, Nawaz R, Al-Hussain SA, Rizwan M, Bukhari A, Waseem M, Irfan A, Inam A, Zaki MEA (2023) Enhancing chromium removal and recovery from industrial wastewater using sustainable and efficient nanomaterial: a review. Ecotoxicol Environ Saf 263:115231. 10.1016/j.ecoenv.2023.115231 37429088 10.1016/j.ecoenv.2023.115231

[48] Luo X, Bai P, Wang X, Zhao G, Feng J, Ren H (2019) Preparation of nitrogen-doped carbon quantum dots and its application as a fluorescent probe for Cr(VI) ion detection. New J Chem 43:5488–5494. 10.1039/C8NJ06305B

[49] Xiao J, Cheng Y, Guo C, Liu X, Zhang B, Yuan S, Huang J (2019) Novel functional fiber loaded with carbon dots for the deep removal of Cr(VI) by adsorption and photocatalytic reduction. J Environ Sci (China) 83:195–204. 10.1016/j.jes.2019.04.008 31221382 10.1016/j.jes.2019.04.008

[50] Luo B, Yang H, Zhou B, Ahmed SM, Zhang Y, Liu H, Liu X, He Y, Xia S (2020) Facile synthesis of luffa sponge activated carbon fiber based carbon quantum dots with green fluorescence and their application in Cr(VI) determination. ACS Omega 5:5540–5547. 10.1021/acsomega.0c00195 32201847 10.1021/acsomega.0c00195 PMC7081637

[51] Sudan S, Kaushal J, Khajuria A (2023) Efficient adsorption of anionic dye (congo red) using copper-carbon dots doped magnetic biochar: kinetic, isothermal, and regeneration studies. Clean Techn Environ Policy. 1–17. 10.1007/s10098-023-02621-0.

[52] Cardoso RMF, Cardoso IMF, da Silva LP, Esteves da Silva JCG (2022) Copper(II)-doped carbon dots as catalyst for ozone degradation of textile dyes. Nanomaterials 12:1211. 10.3390/nano12071211 35407329 10.3390/nano12071211 PMC9003027

[53] Zhu PP, Cheng Z, Du LL, Chen Q, Tan KJ (2018) Synthesis of the Cu-doped dual-emission fluorescent carbon dots and its analytical application. Langmuir 34:9982–9989. 10.1021/acs.langmuir.8b01230 30056723 10.1021/acs.langmuir.8b01230

[54] Liu Y, Wu P, Wu X, Ma C, Luo S, Xu M, Wei L, Liu S (2020) Nitrogen and copper (II) co-doped carbon dots for applications in ascorbic acid determination by non-oxidation reduction strategy and cellular imaging. Talanta 210:120649. 10.1016/j.talanta.2019.120649 31987173 10.1016/j.talanta.2019.120649

[55] Wu J, Yang J, Feng P, Huang G, Xu C, Lin B (2020) High-efficiency removal of dyes from wastewater by fully recycling litchi peel biochar. Chemosphere 246:125734. 10.1016/j.chemosphere.2019.125734 31918084 10.1016/j.chemosphere.2019.125734

[56] Xue N, Kong X, Song B, Bai L, Zhao Y, Lu C, Shi W (2017) Cu-doped carbon dots with highly ordered alignment in anisotropic nano-space for improving the photocatalytic performance. Sol RRL 1:1–8. 10.1002/solr.201700029

[57] Zhuo S, Gao L, Zhang P, Du J, Zhu C (2018) Living cell imaging and sensing of hydrogen sulfide using high-efficiency fluorescent Cu-doped carbon quantum dots. New J Chem 42:19659–19664. 10.1039/c8nj03654c

[58] Lin L, Xiao Y, Wang Y, Zeng Y, Lin Z, Chen X (2019) Hydrothermal synthesis of nitrogen and copper co-doped carbon dots with intrinsic peroxidase-like activity for colorimetric discrimination of phenylenediamine isomers, Microchim. Acta. 186:0–710.1007/s00604-019-3404-y 10.1007/s00604-019-3404-y 30989397

[59] Ren X, Liu J, Ren J, Tang F, Meng X (2015) One-pot synthesis of active copper-containing carbon dots with laccase-like activities. Nanoscale 46:19641–19646. 10.1039/C5NR04685H 10.1039/c5nr04685h 26548709

[60] Xu Q, Wei J, Wang J, Liu Y, Li N, Chen Y, Gao C, Zhang W, Sreeprased TS (2016) Facile synthesis of copper doped carbon dots and their application as a “turn-off” fluorescent probe in the detection of Fe^3+^ ions. RSC Adv 6:28745–28750. 10.1039/c5ra27658f

[61] Zhang Y, Liu J, Wu X, Tao W, Li Z (2020) Ultrasensitive detection of Cr(VI) (Cr_2_O_7_^2−^/CrO_4_^2−^) ions in water environment with a fluorescent sensor based on metal-organic frameworks combined with sulfur quantum dots. Anal Chim Acta 1131:68–79. 10.1016/j.aca.2020.07.026 32928481 10.1016/j.aca.2020.07.026

[62] Li HY, Li D, Guo Y, Yang Y, Wei W, Xie B (2018) On-site chemosensing and quantification of Cr(VI) in industrial wastewater using one-step synthesized fluorescent carbon quantum dots. Sensors Actuators, B Chem 277:30–38. 10.1016/j.snb.2018.08.157

[63] Ma Y, Chen Y, Liu J, Han Y, Ma S, Chen X (2018) Ratiometric fluorescent detection of chromium(VI) in real samples based on dual emissive carbon dots. Talanta 185:249–257. 10.1016/j.talanta.2018.03.081 29759197 10.1016/j.talanta.2018.03.081

[64] Tan Q, An X, Pan S, Liu H, Hu X (2021) Hydrogen peroxide assisted synthesis of sulfur quantum dots for the detection of chromium (VI) and ascorbic acid, Spectrochim. Acta Part A Mol Biomol Spectrosc 247:119122. 10.1016/j.saa.2020.119122 10.1016/j.saa.2020.119122 33161271

[65] Xiao D, Pan R, Li S, He J, Qi M, Kong S, Gu Y, Lin R, He H (2015) Porous carbon quantum dots: one step green synthesis vial-cysteine and applications in metal ion detection. RSC Adv 5:2039–2046. 10.1039/c4ra11179f

